# High‐throughput monitoring of wild bee diversity and abundance via mitogenomics

**DOI:** 10.1111/2041-210X.12416

**Published:** 2015-07-06

**Authors:** Min Tang, Chloe J. Hardman, Yinqiu Ji, Guanliang Meng, Shanlin Liu, Meihua Tan, Shenzhou Yang, Ellen D. Moss, Jiaxin Wang, Chenxue Yang, Catharine Bruce, Tim Nevard, Simon G. Potts, Xin Zhou, Douglas W. Yu

**Affiliations:** ^1^ China National GeneBankBGI‐ShenzhenShenzhenGuangdong518083China; ^2^ Centre for Agri Environmental ResearchSchool of Agriculture Policy and DevelopmentUniversity of ReadingReadingRG66ARUK; ^3^ State Key Laboratory of Genetic Resources and EvolutionKunming Institute of ZoologyChinese Academy of Sciences, KunmingYunnan650223China; ^4^ University of Chinese Academy of SciencesBeijing100094China; ^5^ School of Biological, Biomedical and Environmental SciencesUniversity of HullHullHU67RXUK; ^6^ School of Biological SciencesUniversity of East AngliaNorwich Research ParkNorwichNorfolkNR47TJUK; ^7^ Conservation Grade Ltd.St NeotsCambridgeshirePE196TYUK; ^8^ Charles Darwin UniversityDarwinNTNT0909Australia

**Keywords:** agri‐environment schemes, biodiversity and ecosystem services, farmland biodiversity, genome skimming, Hymenoptera, metabarcoding, metagenomics, mitogenomes, neonicotinoids, pollination

## Abstract

1. Bee populations and other pollinators face multiple, synergistically acting threats, which have led to population declines, loss of local species richness and pollination services, and extinctions. However, our understanding of the degree, distribution and causes of declines is patchy, in part due to inadequate monitoring systems, with the challenge of taxonomic identification posing a major logistical barrier. Pollinator conservation would benefit from a high‐throughput identification pipeline.
2. We show that the metagenomic mining and resequencing of mitochondrial genomes (mitogenomics) can be applied successfully to bulk samples of wild bees. We assembled the mitogenomes of 48 UK bee species and then shotgun‐sequenced total DNA extracted from 204 whole bees that had been collected in 10 pan‐trap samples from farms in England and been identified morphologically to 33 species. Each sample data set was mapped against the 48 reference mitogenomes.
3. The morphological and mitogenomic data sets were highly congruent. Out of 63 total species detections in the morphological data set, the mitogenomic data set made 59 correct detections (93·7% detection rate) and detected six more species (putative false positives). Direct inspection and an analysis with species‐specific primers suggested that these putative false positives were most likely due to incorrect morphological IDs. Read frequency significantly predicted species biomass frequency (*R* ^2^ = 24·9%). Species lists, biomass frequencies, extrapolated species richness and community structure were recovered with less error than in a metabarcoding pipeline.
4. Mitogenomics automates the onerous task of taxonomic identification, even for cryptic species, allowing the tracking of changes in species richness and distributions. A mitogenomic pipeline should thus be able to contain costs, maintain consistently high‐quality data over long time series, incorporate retrospective taxonomic revisions and provide an auditable evidence trail. Mitogenomic data sets also provide estimates of species counts within samples and thus have potential for tracking population trajectories.

## Introduction

Safeguarding wild bee populations and their pollination services is a policy priority (DEFRA 2014; Gilbert 2014) because wild bees play a keystone role in the pollination of wild plants and cultivated crops and thereby help to maintain biodiversity and food production (Breeze *et al*. 2011; Garibaldi *et al*. 2013). However, pollinators are threatened by habitat loss, pesticides, climate change and disease (Potts *et al*. 2010; Goulson *et al*. 2015), and evidence exists of declines in wild pollinators and insect‐pollinated plants (Biesmeijer *et al*. 2006; Cameron *et al*. 2011; Burkle, Marlin & Knight 2013; Ollerton *et al*. 2014), but these analyses use historical records, which suffer from unequal survey effort and geographical bias.

Our understanding of bee population trajectories and responses to conservation interventions could be improved with systematic, comprehensive and auditable monitoring methods (Goulson *et al*. 2015). An important motivation for this work is Lebuhn *et al*.'s (2012) calculation that 200 sampling sites are needed to have a > 90% chance to detect an annual population decline of ≥2% over a 5‐year span. Lebuhn *et al*. estimated that each site would generate 3120 bees per year (pooling 26 biweekly collections), resulting in 3120_bees_x200_sites_x2_yrs1&5_ = 1·25 million bees that need to be identified to species. The total cost was estimated to be US$2 million, assuming that the bees could be identified at a rate of <2 min per specimen. This assumption is possibly heroic, given that wild bee species richness ranges from hundreds to thousands of species per country, many of them difficult to separate morphologically (Schmidt *et al*. 2015).

A large‐scale bee monitoring programme would therefore benefit from a high‐throughput identification pipeline that produces reliable species‐level identifications and estimates of species abundances, is able to add taxa and to incorporate taxonomic revisions (including to already‐processed samples), is robust to sample contamination and staff turnover and is auditable by independent parties. A pipeline that uses high‐throughput DNA sequencing can in principle meet these requirements.

**Figure 1:**
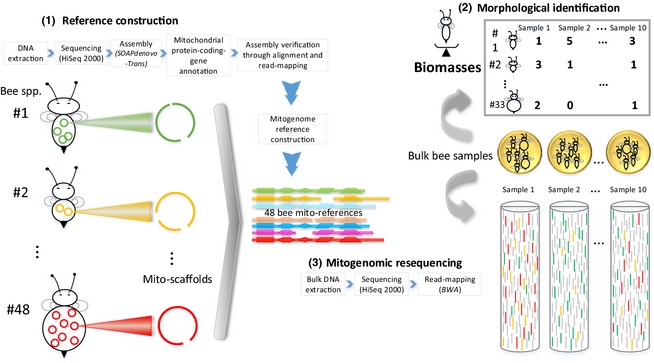
Mitogenomic resequencing pipeline. (1) Reference mitogenomes were assembled from 48 bee species. (2). The 204 bee individuals in 10 bulk samples were morphologically identified to 33 bee species. (3) Total DNA from the same 10 samples was shotgun‐sequenced (the ‘resequencing’ step), and the reads were bioinformatically mapped to the reference mitogenomes, generating Table 1. Note that the vast majority of the output in step 3 was nuclear genome reads, which were discarded.

It is now feasible to assemble large numbers of mitochondrial genomes (mitogenomes), even from species pools (Gillett *et al*. 2014; Tang *et al*. 2014; Andújar *et al*. 2015; Crampton‐Platt *et al*. 2015; Gómez‐Rodríguez *et al*. 2015). Mitogenomes can be thought of as super‐DNA‐barcodes, opening the possibility of ‘mitogenomics’, which we define as the application of bacterial metagenomic methods to the former bacteria now living symbiotically inside eukaryotes. We present a mitogenomics pipeline that shotgun‐sequences total DNA from bulk‐bee samples and conducts taxonomic binning against a reference library of bee mitogenomes (Fig. 1). In our study, bees were first identified morphologically, allowing us to conduct three tests. We asked (1) whether the morphological and mitogenomic data sets detected the same bee species and (2) whether read frequencies could estimate species biomass frequencies. (3) We also conducted community analyses and asked whether the two data sets clustered samples similarly and extrapolated similar estimates of overall bee diversity. We compare and contrast with the output from a metabarcoding pipeline, and finally, we discuss the relative merits of mitogenomics, metabarcoding, quantitative PCR and individual barcoding.

## Materials and methods

### Sampling

Bees were collected as part of a study assessing the effectiveness of agri‐environment schemes for pollinators. Sampling took place in four landscapes in southern England: Chilterns North, Chilterns South, Hampshire Downs and Low Weald. Three farms per landscape were sampled, each in a different agri‐environment scheme. ‘Entry‐Level Stewardship’ is a government‐funded agri‐environment scheme and covered 65% of England's agricultural land in October 2013 (Natural England 2012). ‘Conservation Grade’ is a land‐sparing protocol allocating at least 10% per farm area to wildlife habitat, but allowing some chemical inputs (www.conservationgrade.org, accessed 19 January 2015). ‘Organic’ is a land‐sharing approach, with bans on synthetic chemicals. There were three sampling rounds between 30 April and 23 August 2012. Pan‐trap sampling was used because it is considered the most effective method for sampling bee diversity in European agricultural and grassland habitats and has particular advantages for solitary bees (Westphal, Bommarco & Carré 2008). Pan traps were plastic bowls painted with UV paint to form triplicate sets of blue, white and yellow. Each pan trap was half‐filled with water to which a couple drops of liquid dish soap were added to reduce surface tension. Pan traps were left for 24 h, after which bees were collected and frozen. Bees were defrosted, dried and pinned, then identified to species using the keys of Else (2000) for solitary bees and Prŷs‐Jones & Corbet (2011) for bumblebees. After identification, the bees were returned to the freezer. One individual from each of the 48 most abundant bee species (half the total species richness of 97 species) and the ten largest samples (3–11 species and 13–51 bees per sample) were selected for mitogenomic analysis and shipped in alcohol‐filled tubes to the Kunming Institute of Zoology, China. In one of the ten samples, a single individual of a rare species, *Bombus rupestris*, was present, but since we did not make its reference mitogenome, we omitted this individual from further analysis. All other species in the samples were included in the 48 reference species. For each of the 48 reference bee species, a female not used to make the reference mitogenome was measured for its intertegular (between wing plate) distances, which is correlated with thorax volume, and thus with biomass (Cane 1987).

### Mitogenome assembly

Genomic DNA was extracted from the thorax and legs of each of the 48 reference species following Ivanova, deWaard & Hebert (2006), avoiding the rest of the body to minimise bacterial DNA. A library with an insert size of 200 bp was prepared from each specimen following manufacturer's instruction and sequenced at 2·5 Gb depth and 100 bp PE on an Illumina HiSeq2000 at BGI‐Shenzhen, China. Raw reads were filtered with a Perl script that removes reads containing adaptor contamination (with >15 bp matched to the adaptor sequence), poly‐Ns (>5 bp Ns) or >1% error rate (>10 bp bases with quality score <20) following Zhou *et al*. (2013) and Tang *et al*. (2014). *De novo* assemblies for each bee were generated using *SOAPdenovo‐Trans* (‐K 61) (Xie *et al*. 2014), and scaffolds encoding mitochondrial proteins (mitoscaffolds) were annotated using a custom Perl script described by Zhou *et al*. (2013) with a 774 species reference data base of arthropod mitogenomes (Tang *et al*. 2014), allowing us to remove nuclear mitochondrial insertions (numts). Mitoscaffolds were used to construct bee mitogenome references, which were manually corrected and checked following Tang *et al*. (2014). Each of the 13 mitochondrial protein‐coding genes extracted from the mitoscaffolds, together with reference protein‐coding gene sequences from 6 bees (*Apis cerana, Apis florea, Apis mellifera, Bombus ignitus, Bombus hypocrite sapporensis and Melipona bicolor*), was globally aligned with clustalw 2.1 (Thompson, Higgins & Gibson 1994) and ensured for correct translation frames with mega6 (Tamura *et al*. 2013), allowing us to correct the number of Ns generated during scaffolding of the paired‐end reads. The original reads were then mapped onto the mitoscaffolds with bwa 0.6.2 (Li & Durbin 2009) to identify regions with exceptionally low or zero coverage relative to adjacent regions, and these problematic sites were confirmed or corrected using the *mpileup* command of samtools 0.1.19 (Li *et al*. 2009). Five bee species (*Bombus pratorum, Lasioglossum laevigatum, L. lativentre, L. xanthopus and L. leucozonium*) with relatively poorly assembled mitogenomes were selected for additional sequencing of the remaining limited genomic DNA to improve their assemblies. We pooled the 5 species, prepared a single library of insert size of 500 bp, and sequenced at 2 Gb depth and 300 bp PE on a MiSeq at the Kunming Institute of Zoology. Metagenomic mitoscaffolds from four bee species were assembled as previously described for the HiSeq mitogenome assembly and recovered by blast against their HiSeq mitoscaffolds (*L. laevigatum* sequences were not found), and the longest mitoscaffolds matching by at least 98% identity were used to improve the assemblies.

### Mitogenomic resequencing

From each of the 10 bulk samples, the bees were homogenised in a FastPrep‐24 (MP Biomedicals, Santa Ana, CA, USA), total DNA was extracted using Qiagen DNeasy Blood & Tissue Kits (Hilden, Germany), and 5 μg was used for 250‐bp insert‐size library construction and sequenced at 5–6 Gb depth and 100 bp PE on a HiSeq2000 at BGI‐Shenzhen, China. After data filtering, clean reads from each sample were uniquely mapped using BWA onto the 48 reference mitogenomes at high stringency: 100% read coverage at 99% identity.

For species with incomplete mitogenomes, the number of mapped reads per species and sample was divided by (achieved_mitogenome_length/16000 bp) to derive a normalised read number. Finally, because each reference bee species had been separately sequenced, we could calculate the percentage of reads that were mitochondrial in origin, and we divided the read number per species per sample by this percentage to try to correct for species‐level differences in mitonuclear ratio.

### PCR‐based metabarcoding

We used aliquots of the same DNA extracted from the 10 bulk samples for mitogenomic resequencing and amplified from each a 319‐bp COI fragment, a subunit of the standard COI barcode region. The forward primer was LepF (5′ ATTCAACCAATCATAAAGATATTGG 3′), and the reverse primer (mlCOIintBeeR, 5′ GGDGGRTAWANDGTTCANCCHGTHCC 3′) was modified from mlCOIintR (Leray *et al*. 2013), based on 160 bee COI reference sequences downloaded from GenBank. To build Illumina‐ready PCR amplicons, we attached the standard Illumina HP10 or HP11 sequencing primers, an 8‐bp index sequence, a 0‐ to 5‐bp ‘heterogeneity spacer’ to the 5′ end of LepF, and mlCOIintBeeR (Fig. S3), following Fadrosh *et al*. (2014). Each sample was amplified in three independent reactions and pooled. PCRs were performed in 20 μL reaction volumes containing 2 μL of 10X buffer, 1·5 mM MgCl_2_, 0·2 mM dNTPs, 0·2 μM each primer, 0·6 U Hot Start Taq DNA polymerase (TaKaRa Biosystems, Dalian, China) and approximately 60 ng of genomic DNA. We used a thermocycling profile of 94°C for 3 min: 35 cycles of 94°C for 1 min, 46°C for 1 min and 72°C for 90 s; with a final extension of 72°C for 7 min. PCR products were visualised on 2% agarose gels, gel‐purified using the Qiagen QIAquick PCR purification kit, quantified using the QuantiT PicoGreen dsDNA Assay kit (Invitrogen, Grand Island, New York, USA), pooled and sequenced on a 300‐bp PE Illumina MiSeq run at the Kunming Institute of Zoology. The raw reads were denoised with blue 1.1.2 (‐k 25 ‐g 370) (Greenfield *et al*. 2014), and paired reads were merged in flash 1.2.10 (‐m 10 ‐M 300) (Magoč & Salzberg 2011). The merged reads were split by sample, and the primer sequences and low‐quality reads were removed in the qiime 1.8.0 environment (Caporaso *et al*. 2010) with the script *split_libraries.py* (‐l 330 ‐L 400 ‐H 9 ‐M 4 ‐b 8 ‐r ‐z truncate_remove ‐t –reverse_primer_mismatches 4). Only merged reads with a length of 319 bp were retained, using usearch's 7.0.1090 (Edgar 2010) *sortbylength* command (‐minseqlength 319 ‐maxseqlength 319). These retained reads were clustered into unique sequences in USEARCH with the *derep_fulllength* command, and USEARCH's *UCHIME* function (Edgar *et al*. 2011) was used to perform *de novo* and reference‐based chimera detection and removal, the latter method using the COI sequences of the 48 reference mitogenomes. The remaining sequences were clustered at 98% similarity in crop 1.33 (Hao, Jiang & Chen 2011), producing 468 OTUs, which were assigned taxonomies using the naïve Bayesian classifier (Wang *et al*. 2007) against the 48 COI sequences from the mitogenome data set, using a cut‐off value of 0·8. RDP assigned species‐level taxonomies to 288 of the OTUs, with 232 assigned at a confidence level >0·9. We merged OTUs assigned to the same species and discarded those not assigned to species.

### Analysis

To test whether read number can estimate bee biomasses, we first z‐transformed the normalised read numbers (see Mitogenomic resequencing) and the summed biomasses per sample and then ran a linear regression between read numbers and summed biomasses by species and sample in r's 3.1.2 (R Core Team 2015) base package. Error variance increased with read number, so we re‐analysed using generalised least squares regression in the r package *nlme* 3.1‐118 (Pinheiro *et al*. 2014), and an exponential covariate term (*varExp*) for the biomass frequencies largely removed the heteroscedasticity.

To compare bee communities in the morphological, mitogenomic and metabarcode data sets, we conducted non‐metric multidimensional (NMDS) analyses using *metaMDS()* in the r package *vegan* 2.2‐0 (Oksanen *et al*. 2014). Community dissimilarities were calculated with the *vegdist(method=‘Jaccard’)* function from presence/absence (*binary=TRUE*) and quantitative (*binary=FALSE*) data (read and biomass frequencies). Finally, community correlation was estimated with *vegan*'s Procrustes test: *protest(symmetric=TRUE)*. We also used *vegan*'s *specpool()* function to extrapolate total species richness.

## Results

### Mitogenome assembly

Mitochondrial reads accounted for 0·005–1·319% of each species’ total reads. Of the 48 mitogenomes, 40 were completely assembled with all 13 expected protein‐coding genes, and the other 8 contained 11 or 12 protein‐coding genes (Fig. S1). Mean coverage across all mitogenomes was 224X (range 18·6X‐1855·3X).

### Species detection

**Figure 2:**
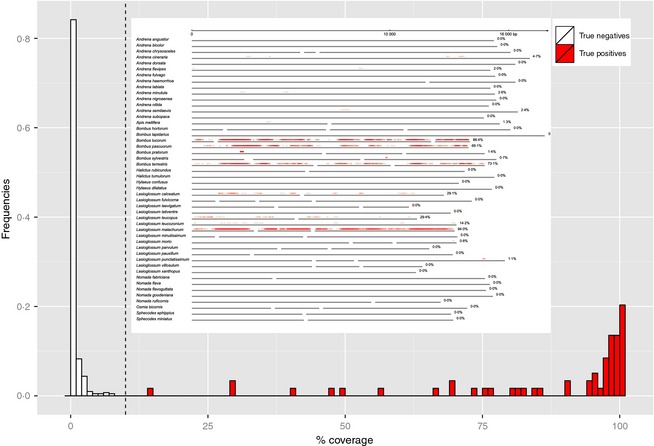
Frequency histograms of read coverages from the true‐negative and true‐positive detections in the mitogenomics pipeline. The dashed line at 10% is the threshold used to calculate species‐detection statistics. Inset: A map of read coverages on the 48 mitogenomes from sample HD_CG_1, showing the 6 true positives (*Bombus pascuorum*,* B. terrestris*,* Lasioglossum calceatum*,* L. leucopus*,* L. leucozonium* and *L. malachurum*) plus *Bombus lucorum*, a putative false positive that was confirmed by species‐specific PCR (Fig. S6).

A total of 204 bees were morphologically identified to 33 species (Table 1). Read coverage per mitogenome was bimodally distributed (Fig. 2), and within mitogenomes, reads mapped approximately evenly (Fig. 2 inset). In order to calculate species‐detection statistics, we classified species as present if read coverage was greater than 10% (see Fig. 2). Using this threshold, mitogenomic resequencing successfully made 59 correct detections out of the 63 species‐sample combinations in the morphological data set (93·7% detection rate for ‘true positives’, mean read coverage 86·7%, range 14·0–100%) and correctly designated 411 species‐sample combinations as absent (‘true negatives’, mean 0·4%, range 0–7·7%) (Table 1). Four species‐sample combinations in the morphological data set were not detected by mitogenomics (putative ‘false negatives’, mean 0·15% read coverage, range 0–0·6%), and 6 species‐sample combinations were detected that were not in the morphological data set (putative ‘false positives’, mean 56·5%, range 12·9–89·9%). Profiling success was 97·9% = (59 + 411)/(59 + 411 + 4+6) (Gómez‐Rodríguez *et al*. 2015).

**Table 1:**
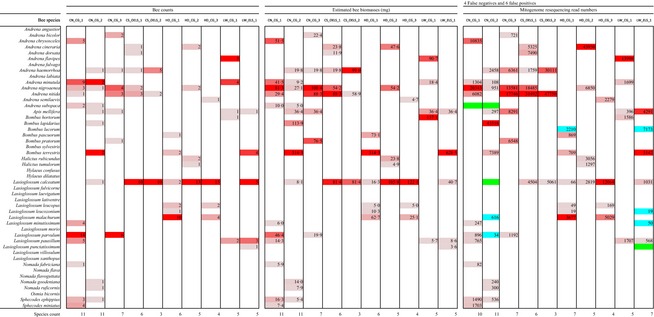
Bee counts, biomasses and mitogenomic resequencing read numbers subdivided by sample (columns) and bee species (rows). To facilitate comparison of samples across the three data sets, each sample (column) is formatted so that the largest number is reddest, descending to light pink. Discrepancies between the morphological data sets (bee counts and biomasses) and the mitogenomic data set are indicated in green (possible false negatives) and blue (possible false positives) in the mitogenomic data set. See Table S3 for the metabarcoding results

PCR‐based metabarcoding was more error‐prone, with 11 false negatives and 49 false positives, compared to 53 true positives (Table S2). Profiling success was 87·5%. Many of the false positives in the metabarcode data set were represented by low read numbers, but the distributions of false‐positive and true‐positive read numbers overlapped (Table S2).

### Biomass frequency vs. read frequency

**Figure 3:**
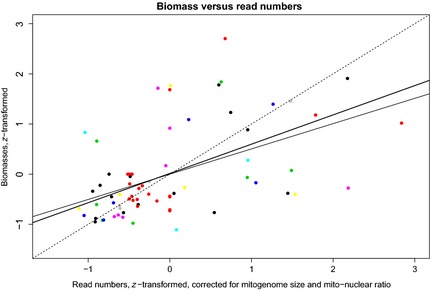
Scatterplot of biomasses versus read numbers. Each data point is one bee species in one sample (samples indicated by colours). The biomass and read numbers were z‐transformed to correct for different sample sizes. The dashed line is the 1:1 line. If all points were on this line, there would be no error in converting from reads to biomass, and thus from biomass to counts (given a species‐typical biomass). The thick solid line is the generalised least squares (GLS) regression (read_freqs ~ 0·0137 + 0·5840*biomass_freqs), and the thin solid line is the linear regression. Both regressions are highly significant (*P* = 0·0001), and the linear regression returns an R^2^ of 24·9%. Conducting the same regression analysis but using metabarcode‐read frequency produced a non‐significant GLS regression (*P* = 0·237, Fig. S4).

*A priori*, larger bees should make up a larger fraction of the total DNA in a sample, and therefore, each species’ mitochondrial‐read frequency should correlate positively with its biomass frequency. We normalised read number per species and sample by each species’ mitogenome size and ratio of mitochondrial to nuclear DNA, which were obtained from our 48 reference bee specimens. Both a linear model and a generalised least squares model to correct for heteroscedasticity indeed found that read‐number frequencies could predict biomass frequencies (*P* < 0·001, *R* ^2^ = 24·9%, statistical details in Fig. 3). Not correcting for mitogenome size and/or mitonuclear ratio reduced explained variance by a few percentage points (*R* ^2^ = 21·0% for uncorrected reads; *R* ^2^ = 21·2% for reads corrected only for mitogenome size). PCR‐based metabarcoding failed to find a biomass–read–number relationship (*P* = 0·237; Fig. S4).

### Community analysis

**Figure 4:**
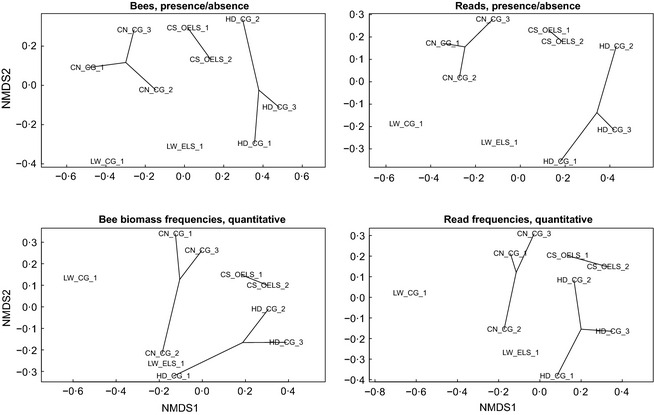
Community analyses. Lines connect samples from the same farm. In the left hand column are the results using the morphological data set (Bee biomass frequencies). In the right‐hand column are the results using the mitogenomic data set (Read frequencies). The top row uses presence/absence. The bottom row uses biomass and read frequencies (quantitative). In general, the morphological and mitogenomic data sets (comparing left with right) organise the samples highly similarly (procrustes *r*_presence/absence_ = 0·981, *P *=* *0·001; *r*_quantitative_ = 0·966, *P *=* *0·001; 9999 permutations). Samples from the same farm and locations tend to cluster together. CN = Chilterns North; CS = Chilterns South, HD = Hampshire Downs, and LW = Low Weald. CG = Conservation Grade farm; OELS = Organic+Entry‐Level‐Stewardship farm; ELS = Entry‐Level‐Stewardship farm. See Fig. S5 for the metabarcoding result.

Comparisons of the morphological and mitogenomic data sets resulted in highly significantly correlated site clusters (statistical details in Fig. 4), with clear groupings by site and region. In contrast, the morphological and metabarcoding ordinations were less similar, and for the presence/absence data, non‐significantly correlated (Fig. S5).

The Chao2 estimator extrapolated similar total species diversities from the two data sets (morphological: 56·3 ± 15·9 SE; mitogenome: 47·9 ± 11·8 SE; Welch's *t*‐test, *t*_d.f.=16·6_ = 0·42, *P *=* *0·68). The metabarcoding data set extrapolated a lower total species diversity (36·0 ± 7·6 SE) due to a lower incidence of singleton species from the many false positives, although given the large standard errors, this extrapolation was also non‐significantly different from the morphological data set (*t*_d.f.=12·9_ = 1·15, *P *=* *0·27).

### Sources of error in species detection

Inspection of the mitogenome data set (Table 1) suggested that all but one of the 10 putative false positives and negatives could reasonably be ascribed to errors in the morphological data set.

Mitogenomic resequencing detected *Bombus lucorum* in two samples (HD_CG_1 and LW_ELS_1, Table 1), but morphology did not. In those samples, *Bombus terrestris* had also been detected by both morphology and mitogenomes, and the workers of these two species are very difficult to tell apart. We designed *B. lucorum*‐specific PCR primers and successfully detected *B. lucorum* in both samples, and we did not detect *B. lucorum* in any other sample (Fig. S6), which suggests that *B. lucorum* in the mitogenomic data set was not a false positive.

We were unable to design PCR tests for the other discrepancies, but in the case of *Andrena subopaca*, which mitogenomics did not detect in two samples (CN_CG_1 and CN_CG_2, green cells, Table 1), multiple individuals of other *Andrena* species were in the same samples. Similarly, mitogenomics did not detect two *Lasioglossum* species in two samples (CN_CG_2 and LW_ELS_1, green cells, Table 1), but both samples had other *Lasioglossum* species that were not detected in the morphological data set (blue cells). *Lasioglossum* species can be difficult to differentiate on the basis of morphology.

Finally, in CN_CG_2, the morphological data set contained only one *Lasioglossum* bee (*L. calceatum*), but the mitogenome data set detected two *Lasioglossum* species. In this case, either mitogenomics truly threw up a false positive, or DNA from two *Lasioglossum* species was in the sample, and mitogenomics detected them both.

## Discussion

We show that mitogenomic resequencing of bulk samples and mapping against a reference data base provides a reliable and high‐throughput method for identifying bee species (Table 1). The output is very suitable for community analysis (Fig. 4) and occupancy modelling, which will allow tracking of changes in species richness and distributions, two of Lebuhn *et al*.'s (2012) three proposed metrics. We note that a higher detection threshold than the 10% we used would have little effect on our classification success, as all ‘true positive’ detections but one had coverages ≥29% (Fig. 2).

Mitogenomic resequencing also successfully recovers quantitative information on biomass frequencies (Zhou *et al*. 2013; Gómez‐Rodríguez *et al*. 2015; Paula *et al*. 2015; Srivathsan *et al*. 2015), although currently, the biomass–read relationship is heteroscedastic and noisy (Fig. 3). Sources of noise include measurement error, bee biomasses that vary across individuals of the same species, especially in social species with workers and queens (Richards & Packer 1996); mitochondrial DNA densities that vary across individuals, tissues within individuals, life spans (Veltri, Espiritu & Singh 1990) and species (this study); and noise introduced during DNA extraction, library construction, sequencing, quality control and read matching. Finally, with an incomplete reference data base, small numbers of reads could be matched to an incorrect species, even when only unique mappings are accepted, as we did.

Nonetheless, even a noisy relationship can be used for tracking the population trajectories of hundreds of bee species at a time. A sample's total bee biomass can be measured before DNA extraction, and after sequencing, the biomass frequencies per species can be converted to absolute biomasses, which can then be converted to counts using species‐specific estimates of biomass per worker bee. Count data produced by mitogenomics will thus contain non‐process (observation) error (Hilborn & Mangel 1997), and the cost of this error is the need for more samples to achieve the same statistical power in detecting population declines. We note that Lebuhn *et al*.'s (2012) simulation did not model taxonomic identification error, which would add a similar sort of error.

In the future, when we have sufficient bee mitogenomes to act as an unbiased reference set, capture‐enrichment techniques (Avila‐Arcos *et al*. 2011) could be employed to increase the proportion of raw mitochondrial reads from the current ~1% to >40% (thus using more of the sequencer output), and we hypothesise that this will reduce heteroscedasticity and increase explained variance in the biomass–read relationship (Fig. 3), by ensuring that high‐biomass species in a sample are represented accordingly. Importantly, low‐biomass species are more reliably represented by fewer reads (Fig. 3), which suggests that low‐abundance species will be identified as such. This is of particular importance, given that low‐abundance species are arguably of greater conservation concern.

In sum, mitogenomics has high potential for allowing monitoring programmes (DEFRA 2014) to track pollinator populations and to assess and target appropriate conservation interventions. Mitogenomics pipelines possess institutional advantages desired in an identification pipeline. Automated taxonomic identification, even for cryptic species, should contain cost inflation, maintain consistently high quality data over long time series and provide an auditable evidence trail, since data sets can be independently analysed at any stage of the bioinformatic pipeline, and, at extra cost, parallel samples can be taken and processed independently. Moreover, taxonomic revisions and new taxa, such as pests, their predators and other pollinators, can be incorporated at any time by (re‐)mapping old and new sequencing data sets against new reference data sets. The mitogenomic pipeline is scalable to more species and larger samples, as we are relying on software and sequencers designed for whole‐genome scale resequencing. The skills needed to carry out a mitogenomics pipeline (non‐destructive DNA extraction, running bioinformatic scripts) are easily learned, with the other steps able to be outsourced to sequencing centres.

A key advantage of mitogenomics is the opportunity to do away with PCR, which reduces laboratory workload, sequence error and contamination risk and therefore results in lower rates of false‐positive and false‐negative species detections relative to metabarcoding (Table 1 vs. Table S2). We observe that despite the fact that the bees in this study were handled for morphological identification and were thus exposed to more cross‐contamination than would be the case in a pure molecular study, read‐coverage values of the true negatives and true positives did not overlap in the mitogenomic data set (Fig. 2), but they did overlap in the metabarcoding data set (Table S2). We conclude that contaminants are inherently easier to detect and omit in a mitogenomic pipeline than in a metabarcoding pipeline. This is a crucial feature in a large‐scale, long‐term monitoring programme where it is impossible to guarantee that collecting and sorting apparatus has always been correctly cleaned between samples.

It is worth emphasising that our 48‐species reference data set deliberately included species that were abundant overall in the landscape but not present in our ten‐sample morphological data set, and thus, we did not expect to detect these species. With the exception of *Bombus lucorum*, which our PCR test suggests was indeed present, these non‐expected species were not detected. This suggests that a synoptic reference data base *per se* will not produce false positives.

It is even possible to run a mitogenomics pipeline without whole mitogenomes. Gómez‐Rodríguez *et al*. (2015) have shown it possible to map against a reference data base of only standard, 5′‐end COI DNA barcodes. However, the advantages of mitogenomes are that the larger target makes resequencing more efficient (Zhou *et al*. 2013; Tang *et al*. 2014; Gómez‐Rodríguez *et al*. 2015; Paula *et al*. 2015), and mitogenomes provide more resolved phylogenetic information (Gillett *et al*. 2014; Andújar *et al*. 2015; Crampton‐Platt *et al*. 2015). Mapping to mitogenomes also increases detection confidence, because only species that are truly present in a sample will produce DNA reads that map across the whole mitogenome (Fig. 2 inset), but any stray PCR amplicons will only map to a single locus. The many thousands of bee species that have been collected for standard DNA barcoding can therefore be used for resequencing, and in the future, these specimens can be used as DNA sources for mitogenome assembly.

### Alternative pipelines

#### Metabarcoding

The big advantage of metabarcoding is that, with appropriate controls and filtering, it can estimate beta and alpha diversity from bulk samples in which taxa are not well characterised and there is no reference data base, such as with meiofauna (Fonseca *et al*. 2010), environmental DNA (Yoccoz *et al*. 2012) and novel locations (Ji *et al*. 2013). The cost is that PCR endpoint read numbers are not reliable estimates of starting DNA concentrations (Fig. S4) (Amend, Seifert & Bruns 2010; Yu *et al*. 2012), due to inherent stochasticity and since each nucleotide mismatch between primer and primer region can result in a ten‐fold drop in amplification (Piñol *et al*. 2014). Amplification bias is what makes it difficult to identify contaminants, because contaminant tissue might match primers better than some of the truly present taxa. We think this is why some of our false‐positive read numbers are greater than some of the true‐positive read numbers (Table S2). Stochasticity in end‐point PCR read numbers can also play a role in amplifying contaminants.

Another challenge for metabarcoding is primer design. We used fusion primers with heterogeneity spacers to make a separate library for each sample, which prevents tag jumping (Schnell, Bohmann & Gilbert 2015) and to increase sequence entropy, which improves sequence quality, but fusion primers are longer and thus somewhat less likely to amplify species, which might have contributed to the greater number of false negatives relative to the mitogenomics data set.

#### qPCR/ddPCR

qPCR (quantitative PCR) and ddPCR (droplet‐digital PCR) can quantify species‐specific DNA concentrations (Doi *et al*. 2015), given properly designed primers and probes, and samples and primer sets can be multiplexed. However, these systems have not yet, to our knowledge, been applied to bulk samples (although they are widely used for environmental DNA (Ficetola *et al*. 2015)), and it remains unclear whether this approach can be scaled up to hundreds or thousands of species. Moreover, adding taxa would require re‐amplification of all samples.

#### Massively parallel barcoding

Surprisingly, the most competitive alternative to mitogenomics could be individual‐based DNA barcoding, in which (portions of) individual bees are separately extracted, amplified and sequenced in parallel using tagged amplicons on Illumina sequencers, at an estimated cost of ≤US$1·5 per specimen (Meier *et al*. 2015; Shokralla *et al*. 2015). Clearly, this method would generate the best count data. However, following Shokralla *et al*.'s (2015) estimate of seven hands‐on hours per 1000 specimens, the 1·25 million bees estimated by Lebuhn *et al*. (2012) would require ~50 person‐months. With mitogenomics, we estimate that 500 samples can be extracted per person‐month, meaning that (26_weeks_ × 200_sites_ × 2_yrs1&5_=) 10 400 samples would require ~21 person‐months before sending to a sequencing centre for library prep and sequencing. In the more seasonal UK, 16 weeks of sampling in 200 sites might generate around 192 000 bees (CQ Tang, pers. comm.), amounting to ~7·7 person‐months for barcoding and ~12·9 person‐months for mitogenomics.

However, barcoding costs scale with specimen number, but mitogenomic costs scale with sample number. Thus, if we include non‐bee taxa (e.g. flies, mites), the mitogenomics workload would not increase, except for the one‐time cost of assembling additional mitogenomes, whereas the individual‐barcoding workload could increase many‐fold.

In conclusion, a mitogenomics approach provides reliable species detection and information on abundance from bulk samples and also provides important institutional advantages: robustness to contamination, the ability to add taxa cheaply and retrospectively, a low skills requirement from staff and the ability to audit samples. Other DNA‐based pipelines do have their advantages, and the choice of which to use will depend on study scale, on‐going improvements in genomics technology, the number of samples that can be pooled per library and the importance of accuracy in specimen counts.

## Author contributions

MT, CJH, YQJ, SHL, TN, SGP, XZ and DWY designed the study, CJH led the fieldwork, and CJH and EDM conducted the morphological identifications. MT led the mitogenome analyses, and YQJ and JXW conducted the metabarcoding analyses. GLM, MHT, SZY, CXY and CB contributed to the field and laboratory analyses, DWY conducted the statistical analyses and wrote the first draft, and all authors contributed to revisions.

## Supporting information


**Fig. S1**. The 48 reference mitogenomes, color‐coded for the 13 protein‐coding genes and the rDNA+Control Region.
**Fig. S2**. Mapping of reads (red line segments) on reference mitogenomes (black lines).
**Fig. S3.** Metabarcoding primers.
**Fig. S4**. Scatterplot of Biomasses versus Metabarcoding Read numbers.
**Fig. S5**. Community analyses for Metabarcoding data.
**Fig. S6**. PCR test for *Bombus lucorum*.

**Table S1.** DNA and assembly quality information for the 48 reference bee species and the 10 bulk samples.
**Table S2.** Bee counts, biomasses, andmetabarcoding read numbers, subdivided by sample (columns) and bee species (rows).

**Appendix S1.** Read map (see Fig. S2) for CN_CG_1.

**Appendix S2.** Read map (see Fig. S2) for CN_CG_2.

**Appendix S3.** Read map (see Fig. S2) for CN_CG_3.

**Appendix S4.**Read map (see Fig. S2) for CS_OELS_1.

**Appendix S5.** Read map (see Fig. S2) for CS_OELS_2.

**Appendix S6.** Read map (see Fig. S2) for HD_CG_1.

**Appendix S7.** Read map (see Fig. S2) for HD_CG_2.

**Appendix S8.** Read map (see Fig. S2) for HD_CG_3.

**Appendix S9.** Read map (see Fig. S2) for LW_CG_1.

**Appendix S10.** Read map (see Fig. S2) for LW_ELS_1.
