# Supplementary material for: High‐throughput monitoring of wild bee diversity and abundance via mitogenomics
Source: Methods Ecol Evol. 2015 Jul 6;6(9):1034–43. doi: 10.1111/2041-210X.12416 (PMC5111398; doi:10.1111/2041-210X.12416)
Supplement: Supplementary file 1 — Fig. S1. The 48 reference mitogenomes, color‐coded for the 13 protein‐coding genes and the rDNA+Control Region. Fig. S2. Mapping of reads (red line segments) on reference mitogenomes (black lines). Fig. S3. Metabarcoding primers. Fig. S4. Scatterplot of Biomasses versus Metabarcoding Read numbers. Fig. S5. Community analyses for Metabarcoding data. Fig. S6. PCR test for Bombus lucorum. [file MEE3-6-1034-s001.docx]

**High-throughput monitoring of wild bee diversity and abundance via mitogenomics: Supplemental information**

Min Tang^*,1^

Chloe J. Hardman^*,2^

Yinqiu Ji^*,3^

GuanliangMeng^1^

ShanlinLiu^1^

Meihua Tan^1,4^

Shenzhou Yang^1^

Ellen D. Moss^5^

Jiaxin Wang^3^

Chenxue Yang^3^

Catharine Bruce^6^

Tim Nevard^7,8^

Simon G. Potts^2^

Xin Zhou^†,1^

Douglas W. Yu^†,3,6^

^1^China National GeneBank, BGI-Shenzhen, Shenzhen, Guangdong 518083China

^2^ Centre for Agri Environmental Research, School of Agriculture, Policy and Development, University of Reading, Reading RG66ARUK

^3^ State Key Laboratory of Genetic Resources and Evolution, Kunming Institute of Zoology, Kunming, Yunnan 650223 China

^4^ University of Chinese Academy of Sciences, Beijing 100094China

^5^School of Biological, Biomedical and Environmental Sciences, University of Hull, Hull HU67RX UK

^6^ School of Biological Sciences, University of East Anglia, Norwich Research Park, Norwich, Norfolk NR47TJ UK

^7^ Conservation Grade, Ltd., St Neots, Cambridgeshire PE196TY UK

^8^ Charles Darwin University, Darwin NT0909 Australia

^*^ These authors contributed equally

^†^Co-corresponding authors: dougwyu@gmail.com; xinzhou@genomics.cn

**Figure S1**. The 48 reference mitogenomes, color-coded for the 13 protein-coding genes and the rDNA+Control Region. Bolded genomes were supplemented with MiSeq data.
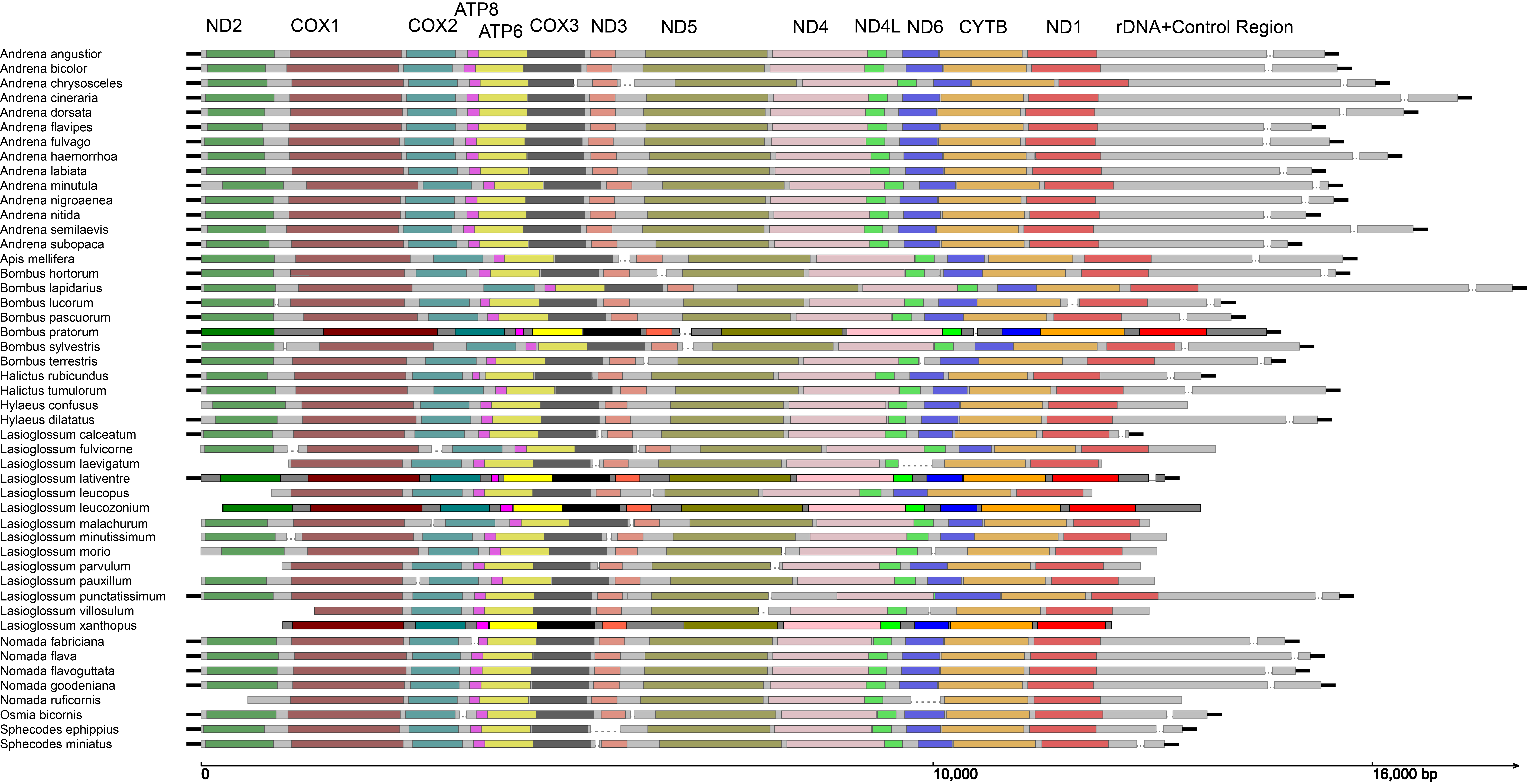


**Figure S2**. Mapping of reads (red line segments) on reference mitogenomes (black lines). Sample shown is CN_CG_1. All ten true positive detections were indicated by a uniform distribution of reads along the corresponding reference mitogenome, with coverage ≥ 40.4%. All true negatives were indicated by low coverage (<5.3%). The one putative false negative (*Andrena subopaca*) was matched by no reads.


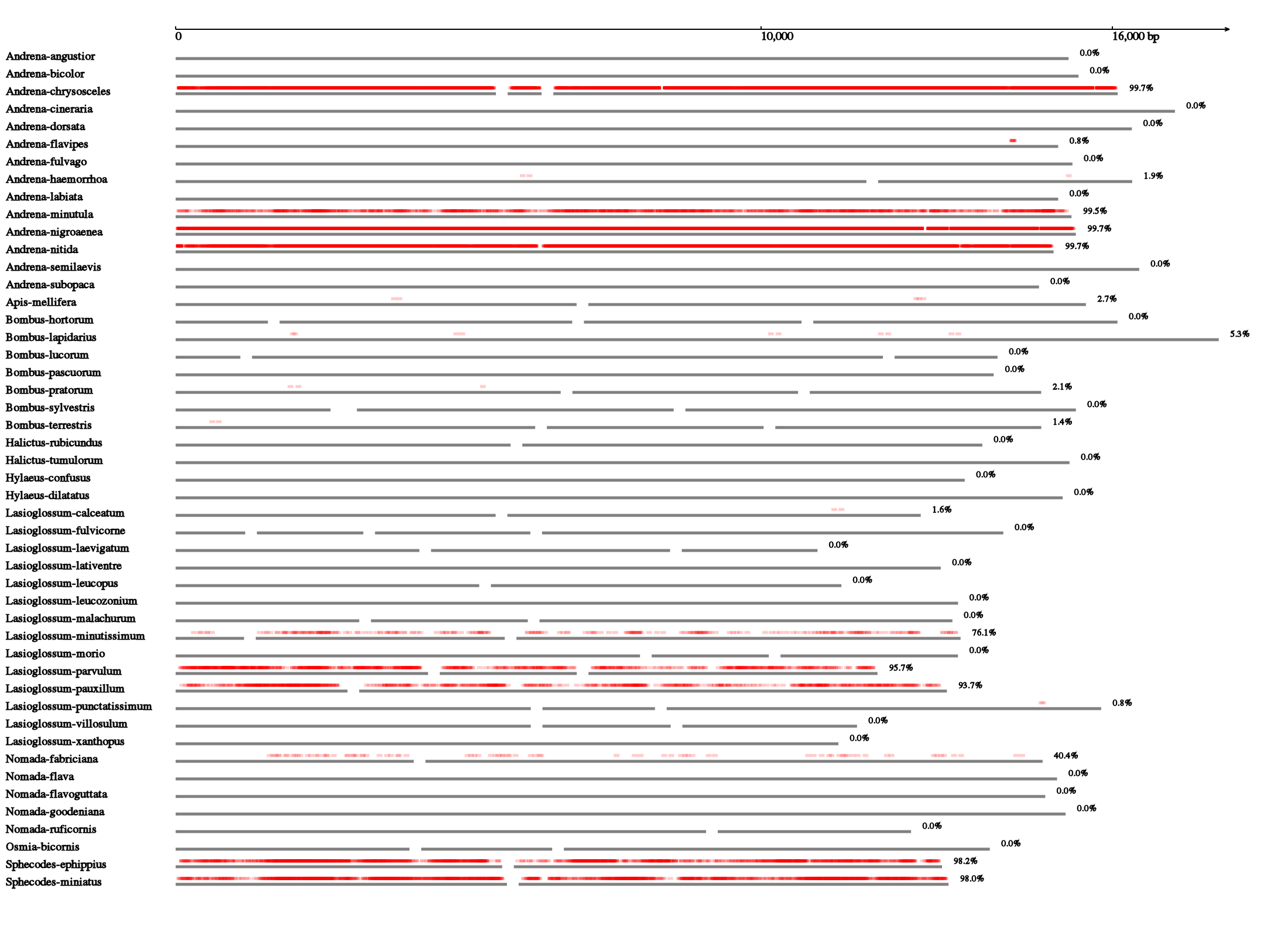


**Figure S3.** Metabarcoding primers.

|  | 5’ HP10/HP11 + index + ***spacer*** + LepF/ mlCOIintBeeR |
| --- | --- |
| LepF-index1 | CAAGCAGAAGACGGCATACGAGATGTGACTGGAGTTCAGACGTGTGCTCTTCCGATCT TCAGTGCG ATTCAACCAATCATAAAGATATTGG |
| LepF-index2 | CAAGCAGAAGACGGCATACGAGATGTGACTGGAGTTCAGACGTGTGCTCTTCCGATCT GTAGCAGA***T*** ATTCAACCAATCATAAAGATATTGG |
| LepF-index3 | CAAGCAGAAGACGGCATACGAGATGTGACTGGAGTTCAGACGTGTGCTCTTCCGATCT ATTCACAG***GT*** ATTCAACCAATCATAAAGATATTGG |
| LepF-index4 | CAAGCAGAAGACGGCATACGAGATGTGACTGGAGTTCAGACGTGTGCTCTTCCGATCT ATTCCATA***CGA*** ATTCAACCAATCATAAAGATATTGG |
| LepF-index5 | CAAGCAGAAGACGGCATACGAGATGTGACTGGAGTTCAGACGTGTGCTCTTCCGATCT TGGCCGAT***ATGA*** ATTCAACCAATCATAAAGATATTGG |
| LepF-index6 | CAAGCAGAAGACGGCATACGAGATGTGACTGGAGTTCAGACGTGTGCTCTTCCGATCT ATGCATAC***TGCGA*** ATTCAACCAATCATAAAGATATTGG |
| mlCOIintBeeR-index1 | AATGATACGGCGACCACCGAGATCTACACTCTTTCCCTACACGACGCTCTTCCGATCT TCAGTGCG GGDGGRTAWANDGTTCANCCHGTHCC |
| mlCOIintBeeR-index2 | AATGATACGGCGACCACCGAGATCTACACTCTTTCCCTACACGACGCTCTTCCGATCT GTAGCAGA***T*** GGDGGRTAWANDGTTCANCCHGTHCC |
| mlCOIintBeeR-index3 | AATGATACGGCGACCACCGAGATCTACACTCTTTCCCTACACGACGCTCTTCCGATCT ATTCACAG***GT*** GGDGGRTAWANDGTTCANCCHGTHCC |
| mlCOIintBeeR-index4 | AATGATACGGCGACCACCGAGATCTACACTCTTTCCCTACACGACGCTCTTCCGATCT ATTCCATA***CGA*** GGDGGRTAWANDGTTCANCCHGTHCC |
| mlCOIintBeeR-index5 | AATGATACGGCGACCACCGAGATCTACACTCTTTCCCTACACGACGCTCTTCCGATCT TGGCCGAT***ATGA*** GGDGGRTAWANDGTTCANCCHGTHCC |
| mlCOIintBeeR-index6 | AATGATACGGCGACCACCGAGATCTACACTCTTTCCCTACACGACGCTCTTCCGATCT ATGCATAC***TGCGA*** GGDGGRTAWANDGTTCANCCHGTHCC |

**Figure S4**. Scatterplot of Biomasses versus Metabarcoding Read numbers. Each data point is one bee species in one sample. Colours indicate the 10 samples. The biomass and read numbers were z-transformed to correct for different sample sizes. The dashed line is the 1:1 line. If all points were on this line, there would be no error in converting from reads to biomass, and thus from biomass to counts (given a species-typical biomass). The solid line is thenon-significant Generalised Least Squares (GLS) regression (p=0.237).

**Figure S5**. Community analyses for Metabarcoding data. Lines connect samples from the same farm. In the left hand column are the results using the morphological dataset (Bee biomass frequencies). In the right-hand column are the results using the metabarcoding dataset (Read frequencies). The top row uses Presence/Absence data. The bottom row uses biomass and read frequencies (Quantitative). The morphological and metabarcoding datasets (comparing left with right) do not organise the samples similarly in the presence/absence datasets (Procrustes *r*_presence/absence_= 0.424, *p*= 0.39, 9999 permutations) but do show a correlation with the quantitative datasets, which is due to true positive detections being having higher numbers of reads, in general (Table S1; *r*_quantitative_= 0.889, *p*=0.001). Samples from the same farm (connected with line segments) and geographic locationstend to cluster together. Codesfor geographic locations are: CN = Chilterns North; CS = Chilterns South, HD = Hampshire Downs, and LW = Low Weald. Codesfor farm management are: CG = Conservation Grade farm; OELS = Organic + Entry-Level-Scheme farm; ELS = Entry-Level-Scheme farm.

**Figure S6**. PCR test for *Bombus lucorum*. The mt-genome sequences of 48 reference bee species were used to design specific primers for *Bombus lucorum* (LucorumF: 5’ GGTTCATCAATAAGTTTAC 3’ and LucorumR: 5’ TATGCTCGTGTATCTACA 3’). The target fragment length is 787 bp (not including primers). The 33 UK bee species that were detected in the mitogenome dataset (including *Bombus lucorum*) and the 10 mixed samples were tested with the *B. lucorum*-specific primers. PCRs were performed in 30 μL reaction volumes containing 3 μL of 10× buffer, 1.5 mM MgCl_2_, 0.2 mM dNTPs, 0.2 μM each primer, 0.9 U ExTaq DNA polymerase (TaKaRa Biosystems, Dalian, China) and approximately 10 ng of genomic DNA. We used a thermocycling profile of 95 °C for 2 min; 35 cycles of 95 °C for 15 sec, 52 °C for 15 sec, 72 °C for 1 min; and a final extension of 72 °C for 10 min. PCR products were visualized on 2% agarose gels.

The two samples for which the mitogenomic dataset detected *Bombus lucorum* are coded here as M6 and M7, and these two samples are the only two that produced PCR product.

Gel figures of the PCR products:


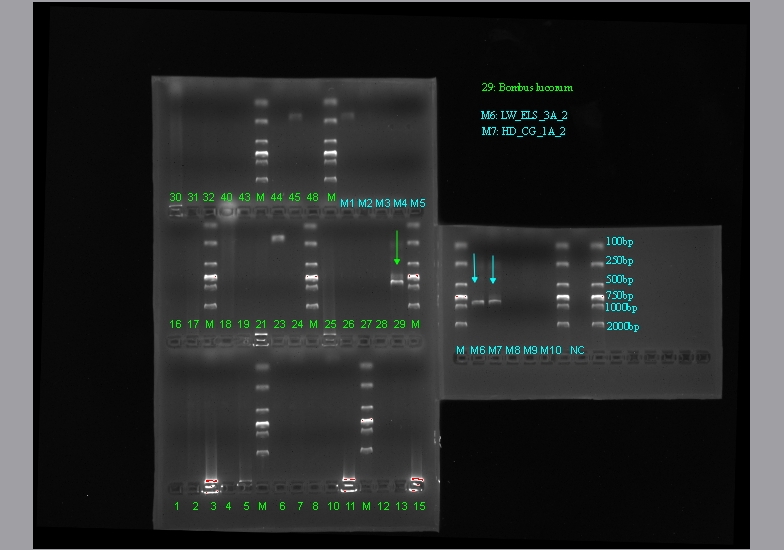


**Table S2**. Bee counts, biomasses, and metabarcoding read numbers, subdivided by sample (columns) and bee species (rows). To facilitate comparison of samples across the three datasets, each sample (column) is formatted so that the largest number is reddest, descending to light pink. Discrepancies between the morphological (Bee counts and Biomass) datasets and the mitogenomic dataset are indicated in green (possible false negatives) and blue (possible false positives) in the mitogenomic dataset.

Label key:

1 - *Lasioglossum calceatum*

2 - *Lasioglossum malachurum*

3 - *Andrena nitida*

4 - *Andrena nigroaenea*

5 - *Andrena cineraria*

6 - *Andrena haemorrhoa*

7 - *Andrena bicolor*

8 - *Andrena minutula*

10 - *Bombus lapidarius*

11 - *Lasioglossum leucopus*

12 - *Andrena dorsata*

13 - *Bombus pascuorum*

15 - *Andrena chrysosceles*

16 - *Lasioglossum pauxillum*

17 - *Lasioglossum parvulum*

18 - *Bombus hortorum*

19 - *Andrena flavipes*

21 - *Halictus rubicundus*

23 - *Lasioglossum minutissimum*

24 - *Bombus terrestris*

25 - *Sphecodesephippius*

26 - *Halictus tumulorum*

27 - *Nomadagoodeniana*

28 - *Nomadaruficornis*

29 - *Bombus lucorum*

30 - *Bombus pratorum*

31 - *Andrena subopaca*

32 - *Andrena semilaevis*

40 - *Nomadafabriciana*

43 - *Lasioglossum leucozonium*

44 - *Sphecodesminiatus*

45 - *Lasioglossum punctatissimum*

48 - *Apismellifera*

M1 – CN_CG_1

M2 – CN_CG_2

M3 – CN_CG_3

M4 – CS_OELS_1

M5 – CS_OELS_2

**M6 – LW_ELS_1 (working code: LW_ELS_3A_2)**

**M7 – HD_CG_1 (working code: HD_CG_1A_2)**

M8 – HD_CG_2

M9 – HD_CG_3

M10 – LW_CG_1

NC – negative control

M – DL2000 marker (TaKaRa Biosystems, Dalian, China)
