# Supplementary figures and images for: High‐throughput monitoring of wild bee diversity and abundance via mitogenomics
Source: Methods Ecol Evol. 2015 Jul 6;6(9):1034–43. doi: 10.1111/2041-210X.12416 (PMC5111398; doi:10.1111/2041-210X.12416)

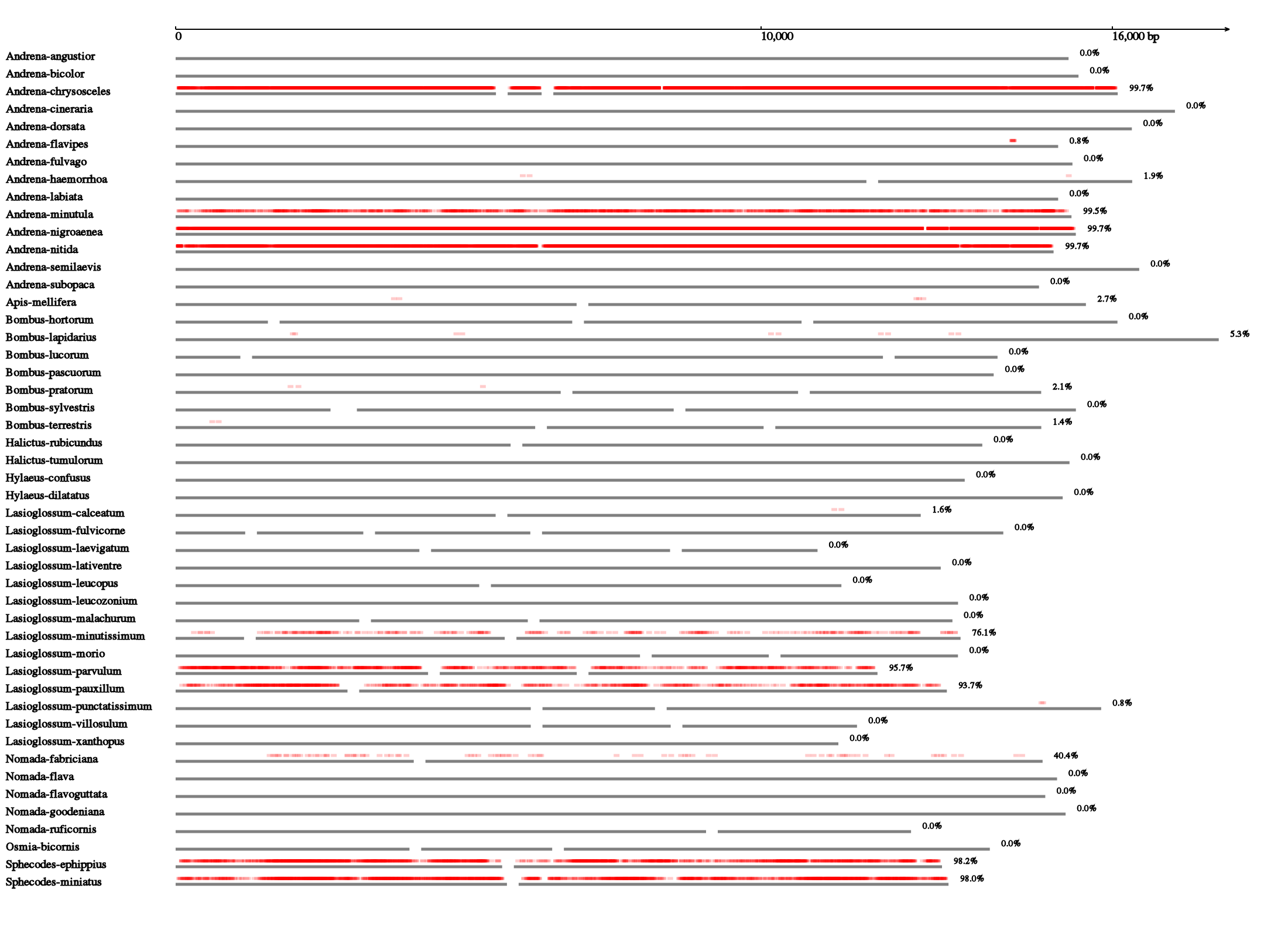

Supplement: Supplementary file 3 — Appendix S1. Read map (see Fig. S2) for CN_CG_1. [file MEE3-6-1034-s003.png]

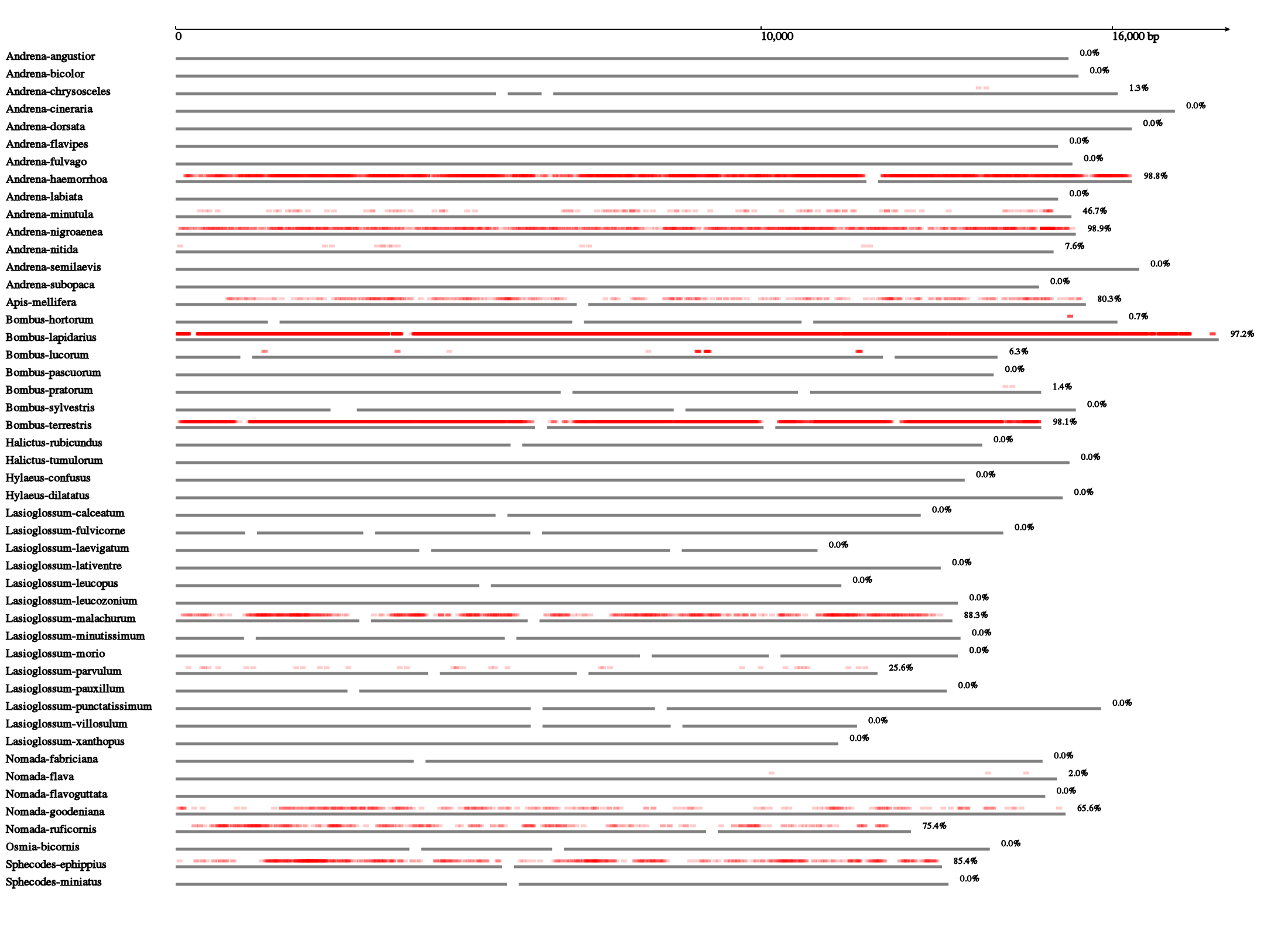

Supplement: Supplementary file 4 — Appendix S2. Read map (see Fig. S2) for CN_CG_2. [file MEE3-6-1034-s004.png]

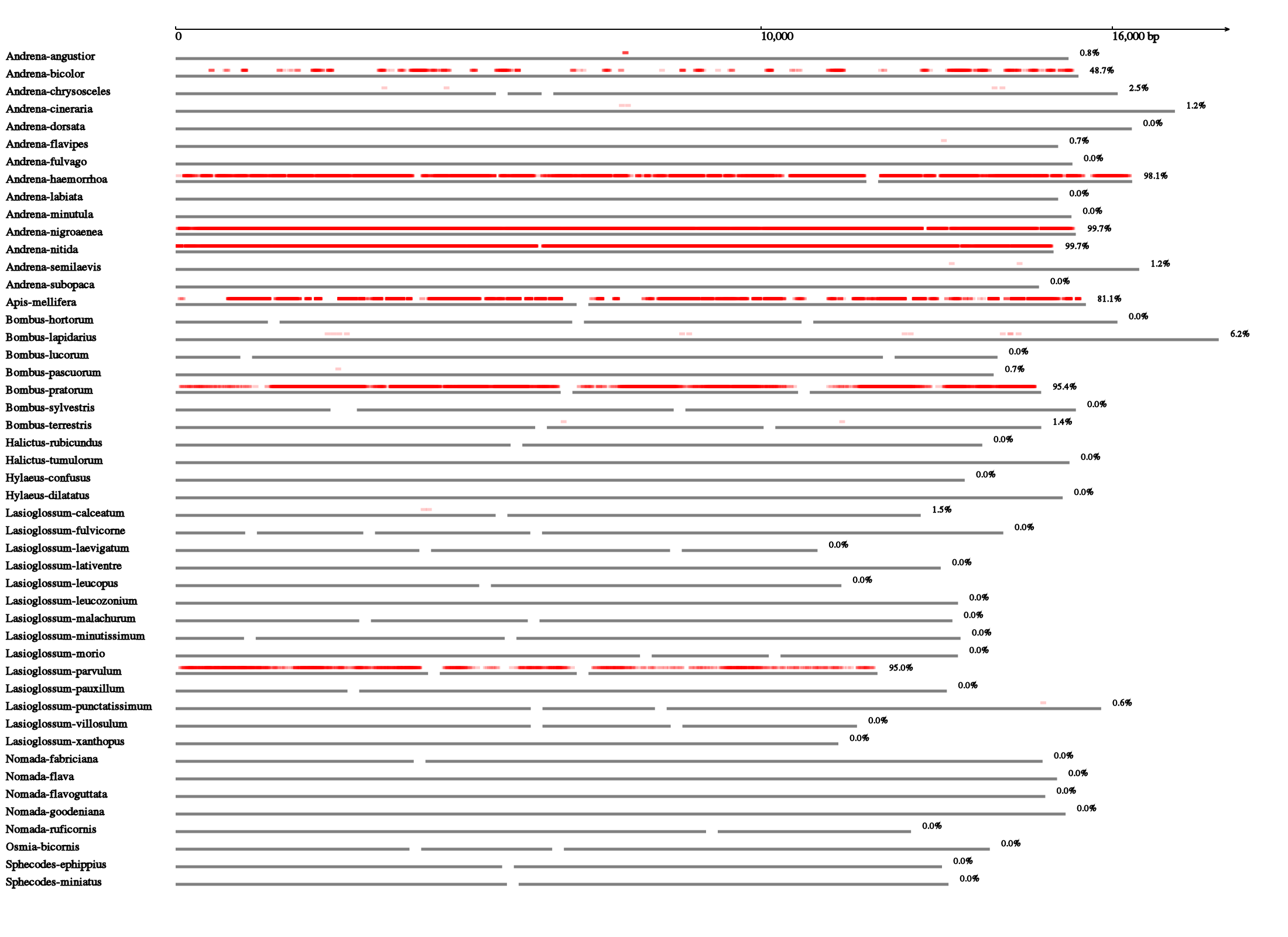

Supplement: Supplementary file 5 — Appendix S3. Read map (see Fig. S2) for CN_CG_3. [file MEE3-6-1034-s005.png]

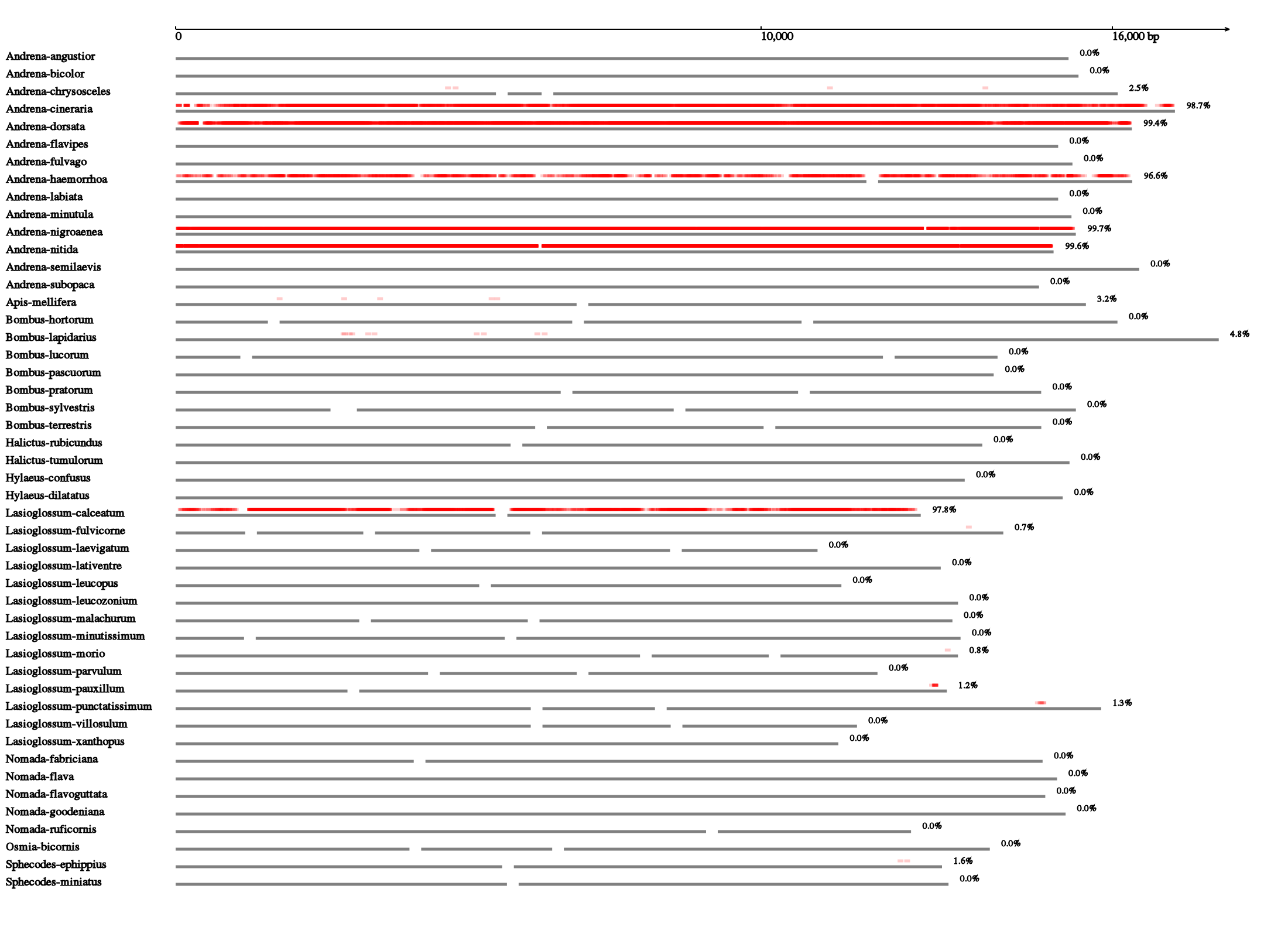

Supplement: Supplementary file 6 — Appendix S4.Read map (see Fig. S2) for CS_OELS_1. [file MEE3-6-1034-s006.png]

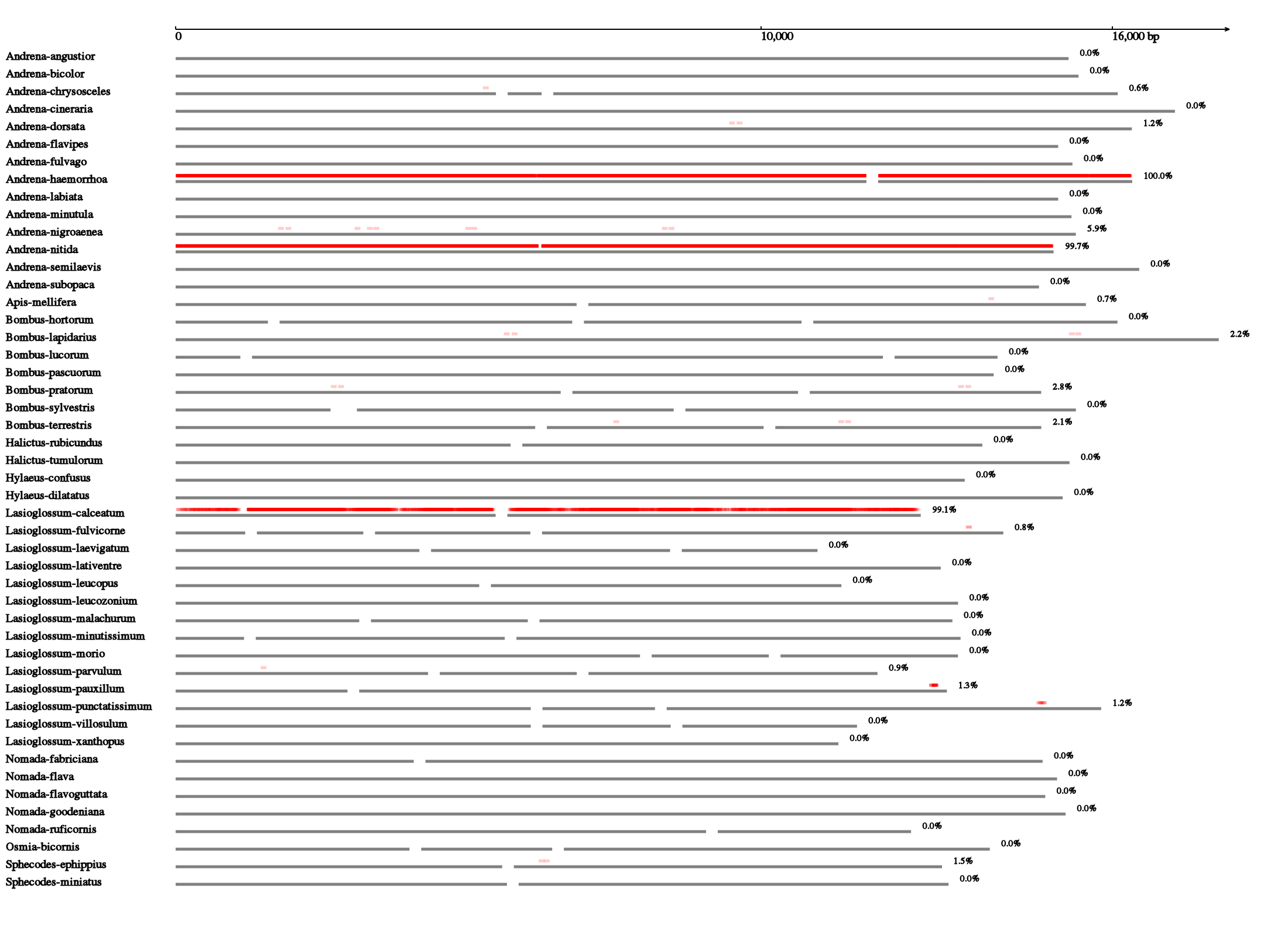

Supplement: Supplementary file 7 — Appendix S5. Read map (see Fig. S2) for CS_OELS_2. [file MEE3-6-1034-s007.png]

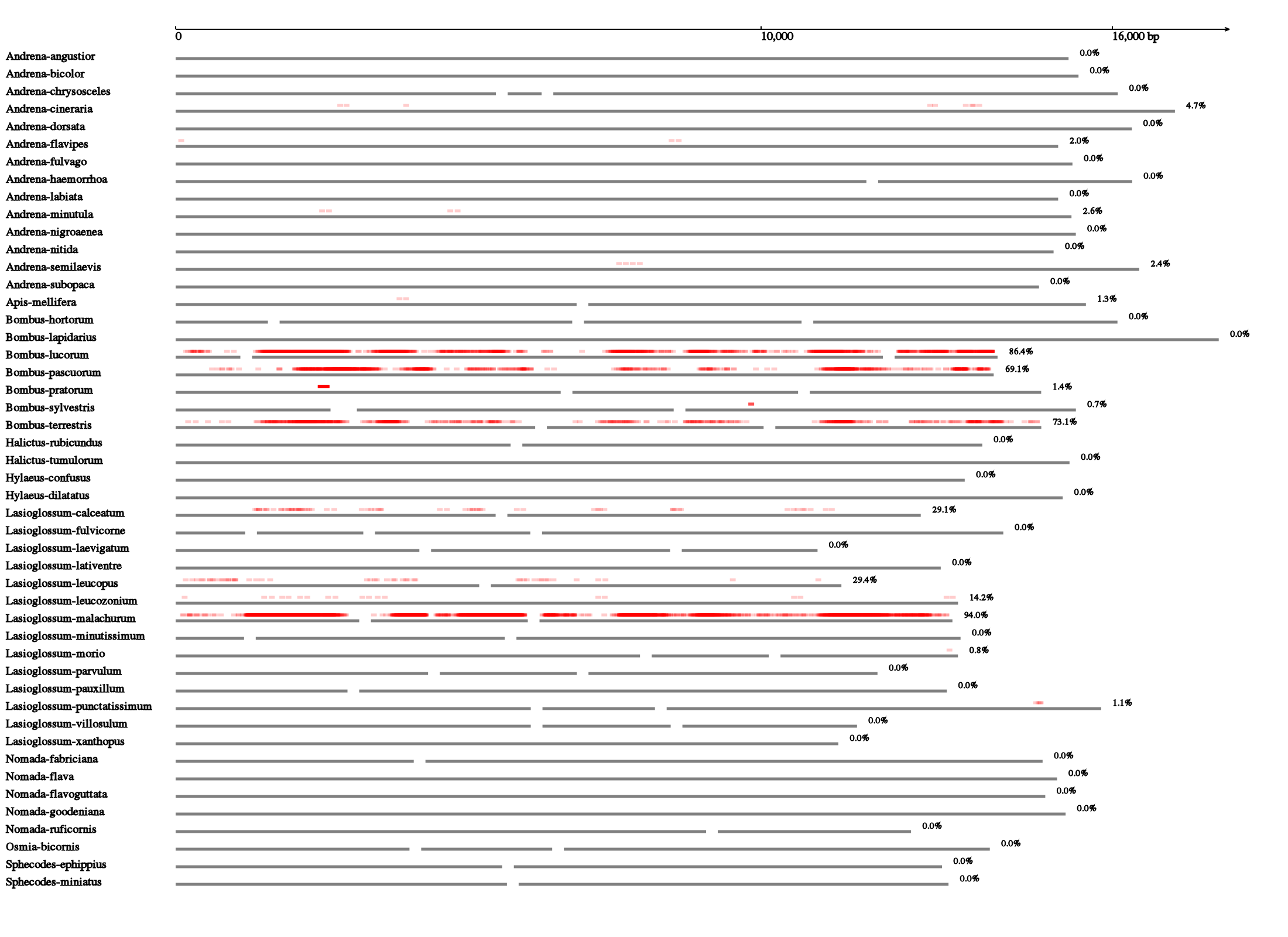

Supplement: Supplementary file 8 — Appendix S6. Read map (see Fig. S2) for HD_CG_1. [file MEE3-6-1034-s008.png]

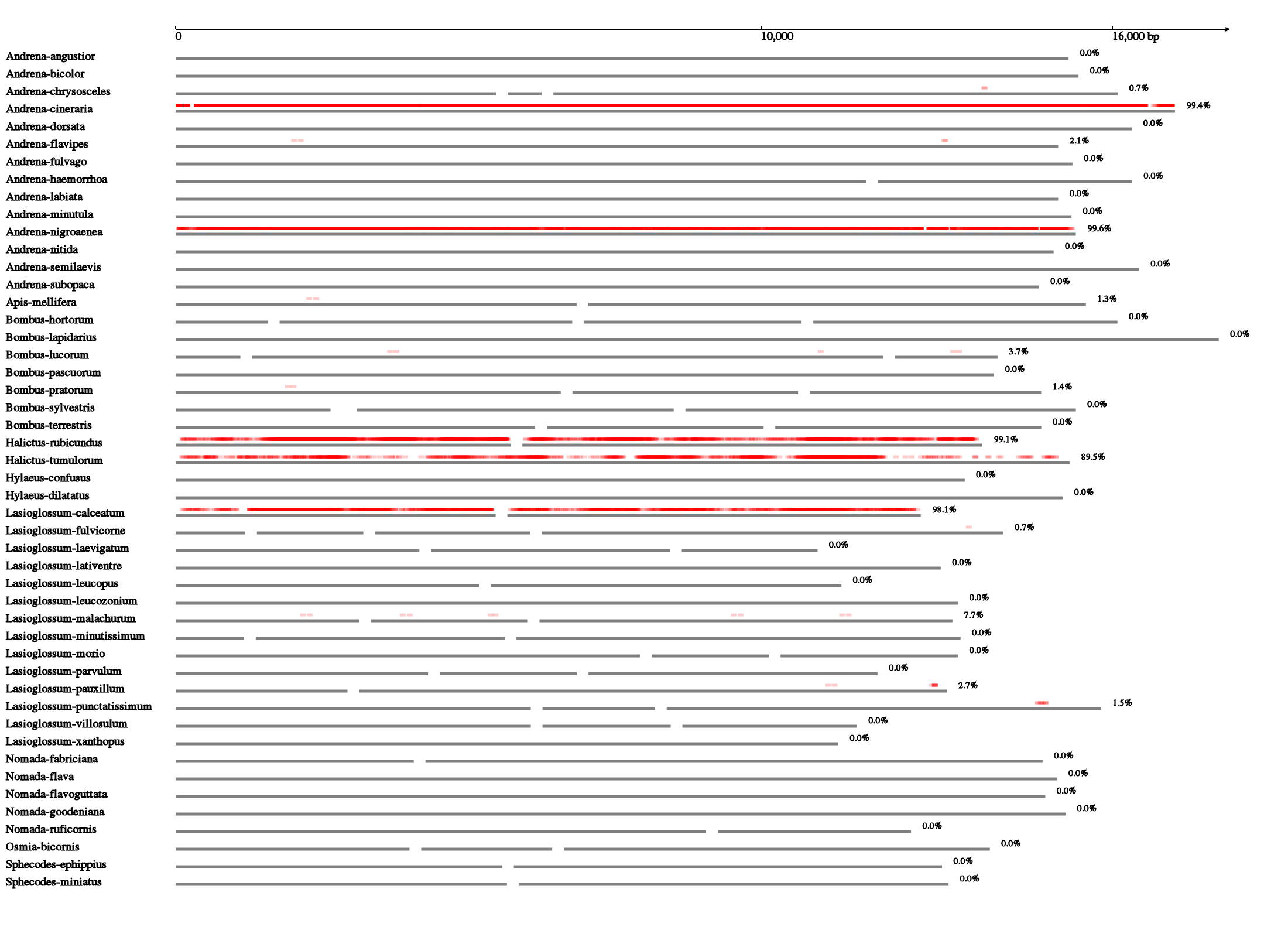

Supplement: Supplementary file 9 — Appendix S7. Read map (see Fig. S2) for HD_CG_2. [file MEE3-6-1034-s009.png]

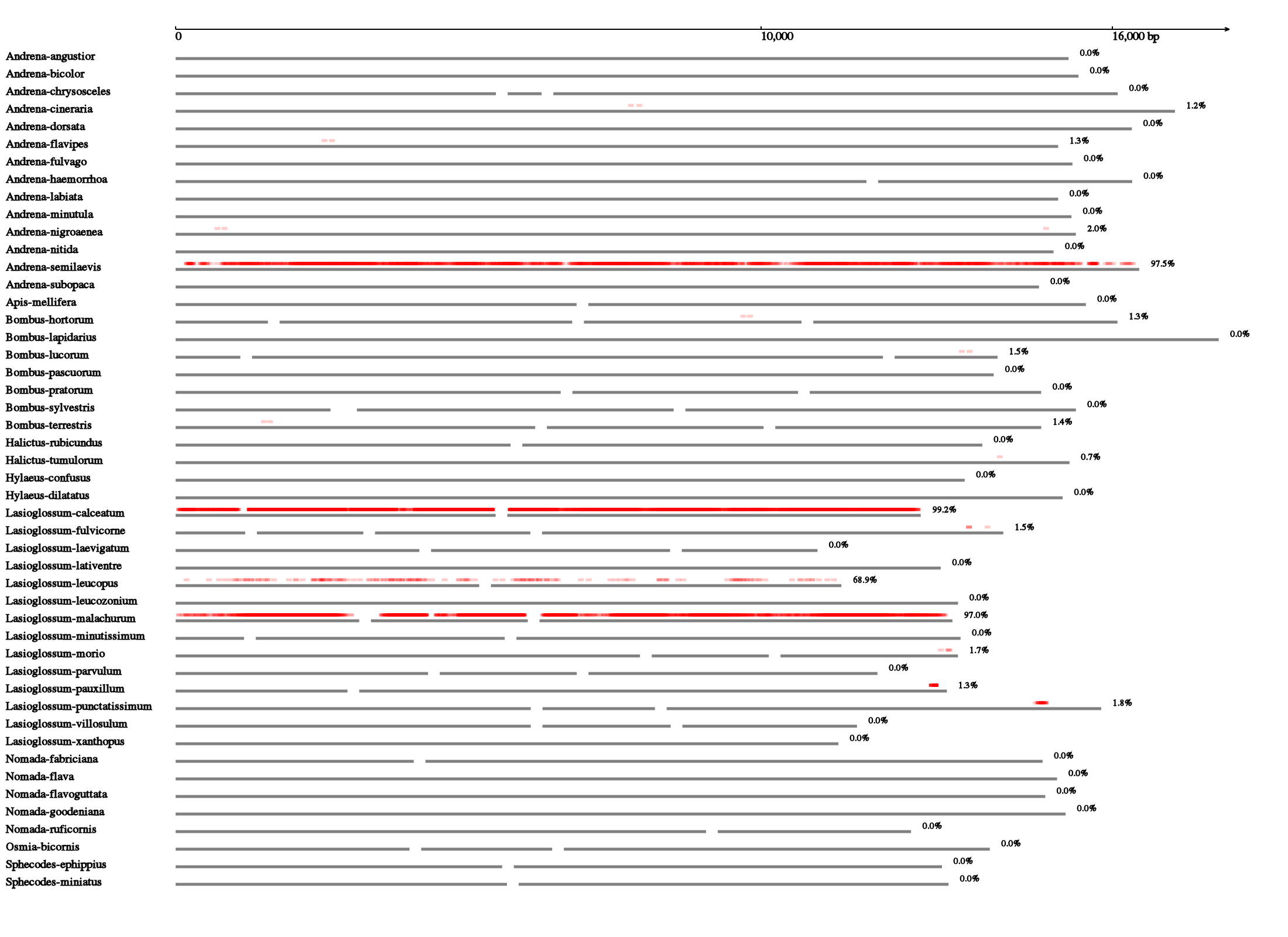

Supplement: Supplementary file 10 — Appendix S8. Read map (see Fig. S2) for HD_CG_3. [file MEE3-6-1034-s010.png]

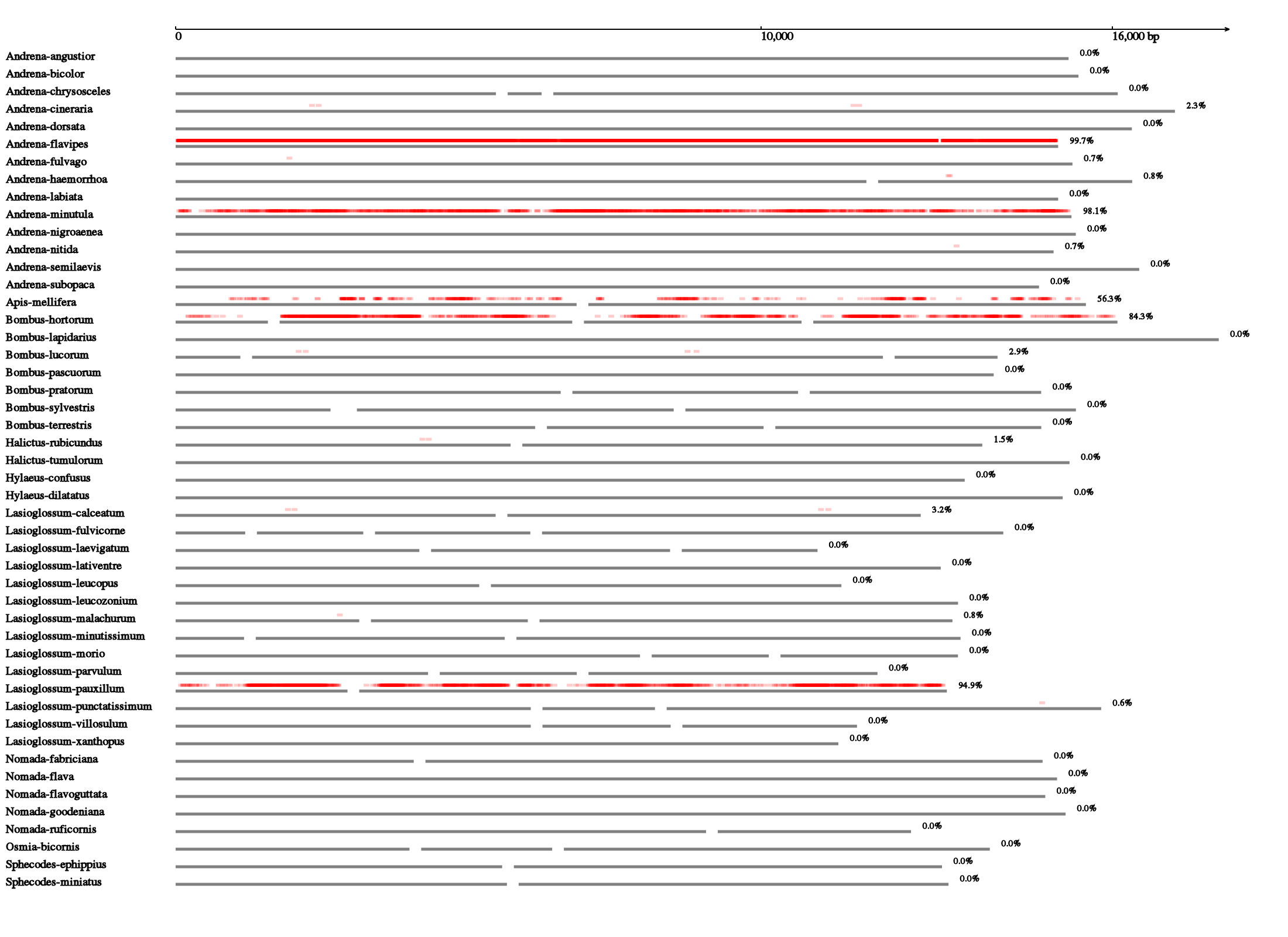

Supplement: Supplementary file 11 — Appendix S9. Read map (see Fig. S2) for LW_CG_1. [file MEE3-6-1034-s011.png]

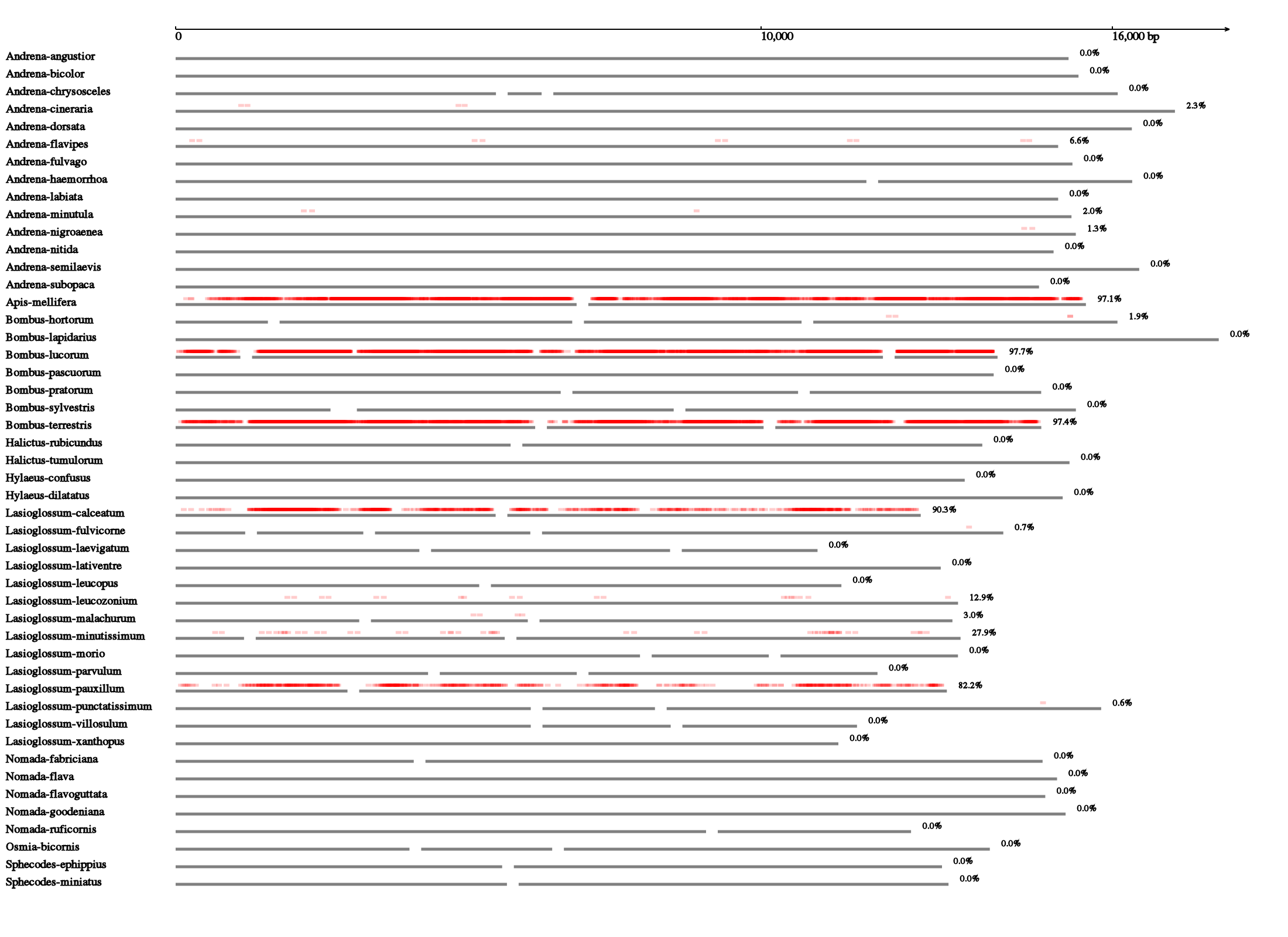

Supplement: Supplementary file 12 — Appendix S10. Read map (see Fig. S2) for LW_ELS_1. [file MEE3-6-1034-s012.png]
